# Integrated Bimodal Fitting for Unilateral CI Users with Residual Contralateral Hearing

**DOI:** 10.3390/audiolres11020018

**Published:** 2021-05-12

**Authors:** Gennaro Auletta, Annamaria Franzè, Carla Laria, Carmine Piccolo, Carmine Papa, Pasquale Riccardi, Davide Pisani, Angelo Sarnelli, Valeria Del Vecchio, Rita Malesci, Elio Marciano

**Affiliations:** 1 Unit of Audiology, Department of Neuroscience, Reproductive Sciences and Dentistry, University of Naples “Federico II”, via Pansini 5, 80131 Naples, Italy; annamaria.franze@unina.it (A.F.); carla.laria@unina.it (C.L.); carmine.piccolo3@unina.it (C.P.); carminepapa85@libero.it (C.P.); nglsarnelli@libero.it (A.S.); valeria.delvecchio@unina.it (V.D.V.); ritamalesci@libero.it (R.M.); marciano@unina.it (E.M.); 2 Advanced Bionics GmbH Max-Eyth Strasse 20, 70736 Fellbach-Oeffingen, Germany; pasquale.riccardi@advancebionics.com; 3 Unit of Audiology, Department of Experimental and Clinical Medicine, Magna Graecia University, Viale Europa, 88100 Catanzaro, Italy; davidepisani@gmail.com

**Keywords:** bimodal system, cochlear implant, hearing aid fitting, APDB

## Abstract

Background: The aim of this study was to compare, in users of bimodal cochlear implants, the performance obtained using their own hearing aids (adjusted with the standard NAL-NL1 fitting formula) with the performance using the Phonak Naìda Link Ultra Power hearing aid adjusted with both NAL-NL1 and a new bimodal system (Adaptive Phonak Digital Bimodal (APDB)) developed by Advanced Bionics and Phonak Corporations. Methods: Eleven bimodal users (Naìda CI Q70 + contralateral hearing aid) were enrolled in our study. The users’ own hearing aids were replaced with the Phonak Naìda Link Ultra Power and fitted following the new formula. Speech intelligibility was assessed in quiet and noisy conditions, and comparisons were made with the results obtained with the users’ previous hearing aids and with the Naída Link hearing aids fitted with the NAL-NL1 generic prescription formula. Results: Using Phonak Naìda Link Ultra Power hearing aids with the Adaptive Phonak Digital Bimodal fitting formula, performance was significantly better than that with the users’ own rehabilitation systems, especially in challenging hearing situations for all analyzed subjects. Conclusions: Speech intelligibility tests in quiet settings did not reveal a significant difference in performance between the new fitting formula and NAL-NL1 fittings (using the Naída Link hearing aids), whereas the performance difference between the two fittings was very significant in noisy test conditions.

## 1. Introduction

Cochlear implants (CIs) are able to successfully restore a sense of hearing in patients with severe-to-profound hearing loss. In recent years, indication criteria for CI candidacy have become less stringent [1], allowing patients with considerable aidable hearing in the contralateral ear to pursue cochlear implantation. These patients typically continue to wear a hearing aid (HA) in the non-implanted ear, resulting in bimodal hearing.

Bimodal hearing has consistently been shown to outperform listening with the CI alone. Benefits have been demonstrated regarding speech intelligibility, sound localization, sound quality, listening effort, and subjective benefit [2,3,4,5,6]. In such studies, typically a variety of HAs are used by the subjects, fitted according to clinical hearing aid fitting practice, independent of the cochlear implant.

To further improve performance in bimodal listeners, Phonak and Advanced Bionics have developed a dedicated bimodal system. The Naída Link Has produced by the Phonak company based in Stafa, Switzerland, and specifically the accompanying bimodal fitting formula (Adaptive Phonak Digital Bimodal (APDB) [7]), have been designed to optimally complement hearing with a CI.

The goal of traditional HA fitting is to optimize speech intelligibility with the HA; frequencies crucial to speech intelligibility (1–4 kHz) are amplified to maximize audibility. In bimodal CI users, especially those with limited contralateral residual hearing, often the CI ear dominates speech intelligibility [3], with the CI by design coding the important frequency region. For such patients, the APDB fitting formula emphasizes audibility of low frequency information complementary to the CI input, which carries temporal fine-structure information to support speech understanding in noise. Additionally, loudness growth functions and automatic gain control (AGC) characteristics are aligned between the Naída CI processor and the Naída Link HA.

Previous research has demonstrated this alignment between CI and HA to be beneficial. Matching the AGC characteristics between the HA and the CI led to improved speech intelligibility in noise tasks using a single competing talker, and the matched AGC was preferred to a standard HA AGC in questionnaires and in a subjective preference test [8].

In this study, speech intelligibility in bimodal CI users upgraded to the Naída Link HA was evaluated in quiet and in noise and compared to results obtained using the previously used HA model as well as the Naída Link HA fitted with the generic NAL-NL1 prescription. The study sought to evaluate not the effect of directional microphones in the noisy environment, but rather, the validity of the APDB formula compared to the NAL, and for this reason, the operating mode of the microphones was set to omnidirectional, and all automated functions were disabled in both the HA and CI.

## 2. Materials and Methods

### 2.1. Subjects

Eleven unilateral CI users with aidable hearing in the contralateral ear participated in this study. They included 6 adults and 5 children, with a mean age of 23 ± 20 years. Four subjects were female. At the time of testing, subjects had on average 5 ± 3 years of experience with their CI and stable maps. All subjects used the Naída CI Q70 sound processor. All subjects had a severe-to-profound hearing loss in the contralateral ear, clinically aided with a GN Resound Enzo or Phonak Naída Sky UP Q70 hearing aid. Detailed subject information is provided in Table 1.

### 2.2. Devices and Fitting

The Adaptive Phonak Digital Bimodal (APDB) fitting formula aims at optimally complementing hearing with the Naída CI by aligning the behavior of the contralateral HA to the Naída CI regarding frequency response, loudness growth functions, and automatic gain control (AGC) characteristics.

In contrast to traditional HA fitting approaches, the APDB fitting formula provides 3 fundamental characteristic points:

- The frequency response is aligned by optimizing low-frequency gain and bandwidth. Low-frequency gain optimization uses the model of effective audibility [7] to ensure audibility of cues that contribute to speech understanding even in relatively quiet environments (55 dB SPL). Depending upon the configuration of the audiogram, this step often results in a gain increase below 1 kHz. This gain increase is limited to make sure that speech at 65 dB SPL does not exceed the most comfortable level. Note that for certain hearing loss configurations (reversed, mild-to-moderate sloping, and flat losses), this gain increase will not be applied. Bandwidth is optimized by ensuring that bandwidth is as wide as possible [9], frequencies between 250 and 750 are audible, and amplification does not extend into dead regions [10];
- Loudness growth is aligned by implementing the input-output function of the cochlear implant in the hearing aid (compression knee points = 63 dB SPL, compression ratios = 12:1);
- The dynamic compression behavior is aligned by porting the Naída CI dual loop AGC into the hearing aid.

Exclusively available for Phonak Naída Link HAs, the APDB fitting formula was used in this study to program a Phonak Naída Link Ultra Power (UP) HA without using any adaptive parameters (directional mics, speech in noise etc.). The patients were wearing their own earmolds, and the output was analyzed by a probe mic.

### 2.3. Test Material

Speech intelligibility was tested using phonetically balanced lists of 20 bisyllabic Italian words [11] in quiet and in competing noise (babble noise). In quiet, speech was presented at 65 dB SPL. For measurements in noise, babble noise (5 male and 5 female talkers) was presented at 65 dB SPL or 70 dB SPL with speech presented at 65 dB, resulting in signal-to-noise ratios (SNRs) of 0 dB and <5 dB, respectively.

Speech was presented from the front (0°) in all test conditions. Noise was presented from 90° and 270°.

### 2.4. Measurement Schedule

Subjects were invited to 2study appointments. At the first appointment, speech intelligibility with their own HAs was measured in quiet and in noise. Subsequently, a Naída Link UP HA was fitted according to each subject’s hearing loss using the APDB fitting formula. At the second appointment following a 7-day acclimatization period to the new HA prescription, speech intelligibility in quiet and in noise was measured with the Naída Link UP HA using the APDB fitting formula and the formula (NAL-NL1) used in their previous HA.

All subjects tried the APDB formula last because the old HAs were already programmed with the NAL-NL1 system.

### 2.5. Statistical Analysis

Statistical analyses were performed using Statistica 12 (TIBCO Software Inc., Palo Alto, CA, USA) with the level of significance set at 0.05. Main effects of noise condition and fitting approach as well as the interaction between them were analyzed using a repeated measures analysis of variance (ANOVA). Post-hoc analyses were performed using Wilcoxon-signed rank tests. Bonferroni corrections for multiple comparisons were applied.

## 3. Results

**Figure 1:**
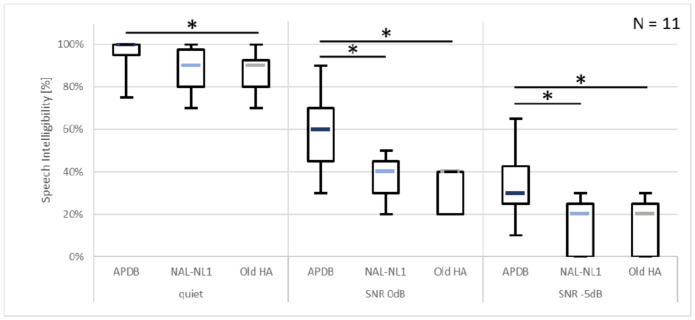
Speech intelligibility scores in % correct for the different noise conditions and different HA models and fitting approaches. Asterisks denote statistical significance.

Median percent correct speech intelligibility is presented in Figure 1 for the three noise conditions (quiet, 0 dB SNR and <5 dB SNR) and the three fitting conditions (Naída Link with APDB, Naída Link with NAL-NL1, and each subject’s own HA). Medians of the individual speech intelligibility improvements for all conditions are listed in Table 2.

Repeated measures ANOVA revealed a statistically significant main effect of the noise condition (F (2, 18) = 386.20, *p* < 0.001), a statistically significant main effect of the fitting condition (F (2, 18) = 57.110, *p* < 0.001), as well as a statistically significant interaction between the two (F (4, 36) = 6.9471, *p* < 0.001).

Post-hoc analysis using Wilcoxon signed rank tests with Bonferroni corrections for multiple comparisons revealed statistically significant differences between the different fitting conditions within each noise condition. The Naída Link HA with APDB yielded significantly better speech intelligibility than the subjects’ own HAs in quiet (Z = 2.52, *p* = 0.035), at 0 dB SNR (Z = 2.80, *p* = 0.015), and at -5 dB SNR (Z = 2.80, *p* = 0.015). Performance with the Naída Link HA was significantly better using the APDB fitting formula that using NAL-NL1 at 0 dB SNR (Z = 2.80, *p* = 0.015) as well as at -5 dB SNR (Z = 2.80, *p* = 0.015).

After conclusion of the study, 10 of the 11 subjects chose to keep the APDB fitting.

## 4. Discussion

Speech intelligibility tests in quiet did not reveal a significant difference in performance between the APDB and NAL-NL1 fittings (using the Naída Link HA) while the performance difference between the two fittings was significant in both noisy test conditions. In quiet, the sensitivity of the test material used was limited by the ceiling effect: using the APDB fitting, the median speech intelligibility score in quiet was 100% with six out of eleven subjects scoring 100%. This may have obscured a benefit of the APDB fitting compared to the NAL-NL1 fitting, which can be seen in the noisy test conditions. More difficult speech material or testing at a lower level could have decreased subject performance to avoid the ceiling effect and therefore might have revealed a significant difference also in the quiet test condition.

Similarly, performance at the most unfavorable SNR of <5 dB was limited by floor effects: five out of eleven subjects scored 0% when using the Naída Link HA with the NAL-NL1 fitting and when using their own HA. However, neither the tests in quiet nor the tests at the more favorable SNR condition of 0 dB revealed a statistically significant difference between the Naída Link NAL-NL1 fitting and their own HA; therefore, the ceiling effect likely did not obscure a significant difference at <5 dB SNR, either.

Previous studies investigating speech intelligibility of bimodal CI listeners revealed equal performance between the dedicated bimodal APDB fitting and the generic HA fitting formulae NAL-NL2 and DSLv5 [11,12] when tested on the same HA. In the study presented here, however, performance with the APDB fitting was found to be significantly better than performance with the generic NAL-NL1 fitting. While the generic HA fitting formulae differed between all three studies, this discrepancy with published literature should be considered nonetheless. One possible explanation lies in the 7-day acclimatization period included in the study protocol. During this acclimatization period, subjects wore the study HA (Naída Link UP) fitted with the APDB fitting formula. At the time of testing, the subjects were therefore partially acclimatized to the APDB fitting but not at all to the NAL-NL fitting, likely resulting in better performance using the APDB fitting. While at only 7 days, the acclimatization period was rather short, the acclimatization effect cannot be disregarded as a possible reason for the performance difference between the APDB and NAL-NL1 fittings.

An additional analysis of the signal audibility, based on the group-average hearing loss, revealed a difference in audibility between the APDB and NAL-NL1 fittings of 8 dB for frequencies below 1 kHz, offering a plausible explanation for the superior performance of the APDB fitting in this subject group. In previous studies [11,12], the group average hearing loss was less severe than in the current study cohort, resulting in lower gain prescriptions of APDB and subsequently a smaller audibility benefit compared to established fitting formulae.

Compared to each subject’s own HA fitted with a generic fitting formula, the Naída Link HA fitted with the APDB formula also resulted in superior speech intelligibility performance. Acclimatization to each HA would in this case favor the subject’s own, clinically used HA with a long period of acclimatization before testing over the Naída Link HA with only 7 days of acclimatization before testing. However, the Naída Link UP provided higher output levels than the clinically used HAs (GN Resound Enzo or Phonak Naída Sky UP Q70), potentially explaining the superior speech intelligibility outcomes. Additionally, the Naída Link HA was fitted for each subject within the study protocol, based on current audiograms, whereas the fitting of their own HA was based on less recent audiogram data and therefore may not have been adequate anymore.

Overall, the superior performance with the APDB fitting may also have been affected by the test order: APDB was always tested last within the test session. If any training effects occurred, they were in favor of the APDB fitting.

After the study, 10 out of 11 subjects continued using the APDB fitting formula as their new clinical provision. The remaining subject, despite better performance with APDB, preferred to return to the previous fitting formula as a result of a more pleasing sound, in his opinion, whereas no one else preferred to resume using their previous hearing aid. The number of patients participating in the study did not allow us to break down the results according to age; in general, it did not appear that the results depended on the age of the subject.

## 5. Conclusions

The dedicated bimodal fitting formula APDB provided statistically significant speech intelligibility benefits over the generic NAL-NL1 fitting formula, especially in challenging listening situations. Most of the subjects involved in the study preferred to continue using the APDB for the fitting of hearing aids.

## Figures and Tables

**Table 1:** Detailed demographic information.

ID	Age (years)	Etiology	Age at Diagnosis (years)	Duration of CI Use (years)	PTA (dB)	Previous HA	Previous Fitting Formula
S01	23	Rubella	2	6	96.25	GN Resound Enzo	NAL-NL1
S02	23	Rubella	2	9	96.25	GN Resound Enzo	NAL-NL1
S03	9	Usher	1	1	81.25	Phonak Naida Sky UP Q70	NAL-NL1
S04	34	Connexin 26	4	2	85	Phonak Naida Sky UP Q70	NAL-NL1
S05	12	Unknown	4	3	97.5	Phonak Naida Sky UP Q70	NAL-NL1
S06	6	Connexin 26	2	2	95	Phonak Naida Sky UP Q70	NAL-NL1
S07	28	Unknown	3	8	107.5	Phonak Naida Sky UP Q70	NAL-NL1
S08	77	Noise	unknown	3	105	GN Resound Enzo	NAL-NL1
S09	10	Connexin 26	2	3	91.25	Phonak Naida Sky UP Q70	NAL-NL1
S10	10	Connexin 26	1	6	101.25	Phonak Naida Sky UP Q70	NAL-NL1
S11	20	Unknown	2	10	103.75	Phonak Naida Sky UP Q70	NAL-NL1

**Table 2:** Median of the individual speech intelligibility improvements. Each entry represents the improvement of the fitting condition indicated in the respective row over the fitting condition indicated in the respective column.

	Quiet		0 dB SNR		−5 dB SNR	
	NAL-NL1	Own HA	NAL-NL1	Own HA	NAL-NL1	Own HA
APDB	5%	5%	20%	30%	20%	20%
NAL-NL1		0%		10%		0%

## Data Availability

The data will be available on the website of the audiology unit www.audiologia.unina.it (accessed date 11 May 2021) after the publication of the article.
